# The Effects of Urban Warming on Herbivore Abundance and Street Tree Condition

**DOI:** 10.1371/journal.pone.0102996

**Published:** 2014-07-23

**Authors:** Adam G. Dale, Steven D. Frank

**Affiliations:** North Carolina State University, Raleigh, North Carolina, United States of America; DOE Pacific Northwest National Laboratory, United States of America

## Abstract

Trees are essential to urban habitats because they provide services that benefit the environment and improve human health. Unfortunately, urban trees often have more herbivorous insect pests than rural trees but the mechanisms and consequences of these infestations are not well documented. Here, we examine how temperature affects the abundance of a scale insect, *Melanaspis tenebricosa* (Comstock) (Hemiptera: Diaspididae), on one of the most commonly planted street trees in the eastern U.S. Next, we examine how both pest abundance and temperature are associated with water stress, growth, and condition of 26 urban street trees. Although trees in the warmest urban sites grew the most, they were more water stressed and in worse condition than trees in cooler sites. Our analyses indicate that visible declines in tree condition were best explained by scale-insect infestation rather than temperature. To test the broader relevance of these results, we extend our analysis to a database of more than 2700 Raleigh, US street trees. Plotting these trees on a Landsat thermal image of Raleigh, we found that warmer sites had over 70% more trees in poor condition than those in cooler sites. Our results support previous studies linking warmer urban habitats to greater pest abundance and extend this association to show its effect on street tree condition. Our results suggest that street tree condition and ecosystem services may decline as urban expansion and global warming exacerbate the urban heat island effect. Although our non-probability sampling method limits our scope of inference, our results present a gloomy outlook for urban forests and emphasize the need for management tools. Existing urban tree inventories and thermal maps could be used to identify species that would be most suitable for urban conditions.

## Introduction

Trees provide ecosystem services that mitigate the negative effects of urban habitats on human and environmental health [1]–[3]. For example, trees reduce urban temperatures, filter air, fix carbon, and reduce energy use through photosynthesis, transpiration, and by providing shade [1], [4]. Unfortunately, many herbivorous pests are more abundant and damaging on urban trees than in natural habitats [5]–[8]. In addition, abiotic stress such as heat and drought are often more severe in urban habitats [8]–[10]. Individually, herbivory or abiotic stress can reduce tree photosynthesis, growth, and survival [11]–[15]. Although not as well documented, these stresses likely occur in concert with one another and combine to reduce tree health and the services trees provide.

Heat is a ubiquitous abiotic stress within urban habitats. Cities can be up to 10°C warmer than surrounding rural areas due to impervious surface cover, anthropogenic heat sources, and low vegetation cover [9], [16]. Warmer temperatures increase vapor pressure deficits [17], which create greater atmospheric demand for water via transpiration [18], [19]. Furthermore, heat and impervious surfaces reduce soil moisture, limiting the amount of water available to roots [17], [20]. Thus, greater transpiration demand may not be met because warmer temperatures and dry soils can also limit stomatal conductance [19], [21]. Therefore, trees stressed by heat and water deficits may not grow optimally, which limits the services they can provide.

Warmer temperatures also increase herbivorous pest abundance on urban trees by increasing insect fecundity and survival [22], [23]. Dale and Frank [23] found that an armored scale insect, *Melanaspis tenebricosa* (Comstock) (Hemiptera: Diaspididae), produced 52% more eggs per adult female with a 1.6°C increase in average site temperature. This contributed to three orders of magnitude more scale insects on street trees at warmer urban sites. Herbivore feeding, particularly by sap-feeding insects, increases water stress [24], and reduces tree photosynthesis [11], growth [13], [14], and aesthetic quality [25]. Thus, on trees that are already stressed by warmer urban temperatures, herbivore feeding may further reduce plant vigor and services [15].

Our hypothesis was that urban heat increases *M. tenebricosa* abundance and that these two factors concurrently increase tree water stress and reduce tree condition. To test this hypothesis, we first determined how temperature affects *M. tenebricosa* abundance on red maple (*Acer rubrum*) street trees. To determine how temperature and pest abundance affect tree stress and growth, we measured tree midday water potential, DBH, and branch growth at various levels of scale insect abundance and temperature. Lastly, we used a standardized method for rating tree condition and a citywide tree inventory to compare trees at various levels of scale insect abundance and temperature within the city. Understanding how urban conditions reduce street tree condition is essential to our understanding of these ecosystems and managing their resources.

## Methods

### Study System: *A. rubrum* and *M. tenebricosa*

Maple trees are among the most common street trees in North Carolina (Personal observation) and the eastern United States [26]. Red maples are deciduous, bottomland tree species indigenous to the eastern United States [27]. Gloomy scale, *M. tenebricosa,* is a native armored scale insect that lives on the trunk and branches of maple trees, where it feeds on cambial parenchyma cells [28]. Heavily infested trees exhibit darkened, discolored bark, twig and branch dieback, and eventually die [7] (Figure S1). These scale insects are more abundant on urban than rural red maple trees, and in the southeastern U.S. have been the most important pest of red maple street trees for over a century [7], [25].

### Study Sites

**Figure 1:**
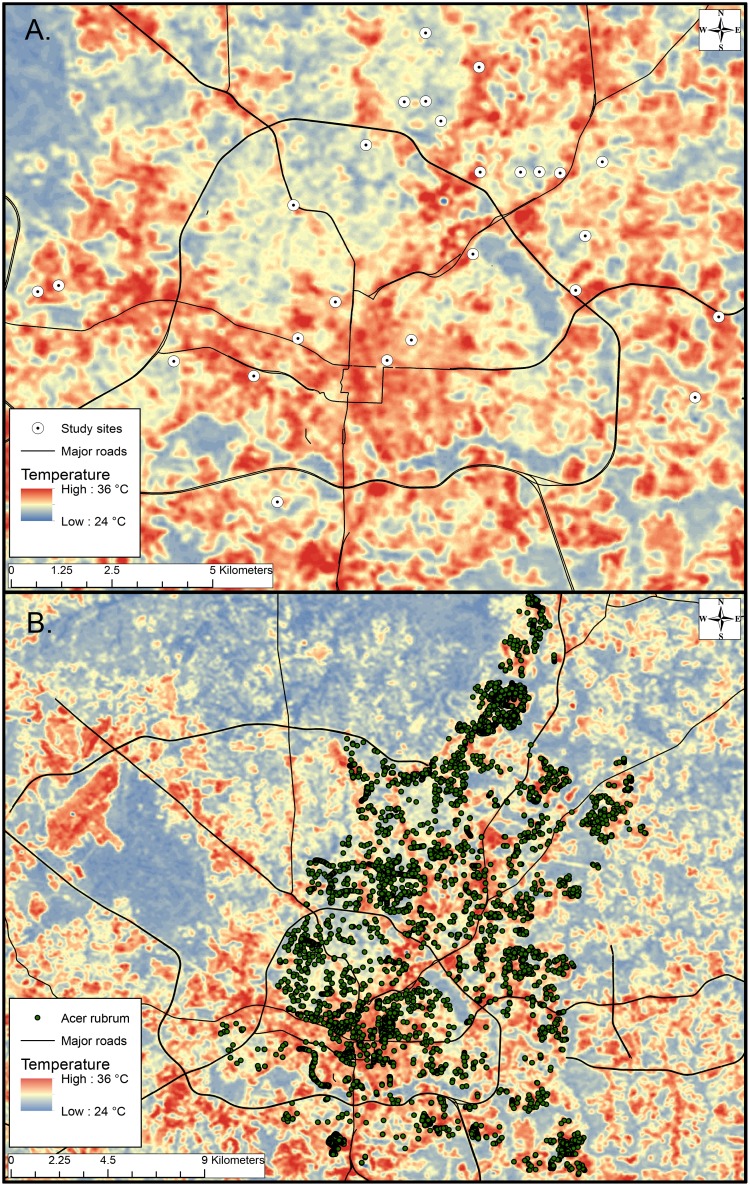
Thermal map of Raleigh, NC on August 18 2007. Temperature ranges from 24 to 36°C designated by the darkest blue and red regions, respectively. (a) Study site selection for *M. tenebricosa* abundance survey, water potential measurement, growth measurements, and condition rating. (b) City-wide red maple street tree dataset of over 8000 trees overlaid on a surface temperature thermal image of Raleigh, NC.

We conducted field studies on red maple trees at 26 sites throughout the Raleigh, NC metropolitan area. All study trees were property of the city of Raleigh, NC. We were granted permission to conduct research on these trees by the Raleigh Parks, Recreation, and Human Resources department. We used a GIS street tree inventory map of city-owned red maples (provided by Raleigh Parks, Recreation, and Human Resources) throughout Raleigh, NC in ArcMap (ArcGIS Desktop 10, Redlands, CA). Sites were selected using a Landsat thermal image acquired on 18 August 2007, prepared as described in [22] and overlaid with the tree inventory map (Figure 1b). The thermal map illustrates variation in surface temperature throughout the city, ranging from 24 to 36°C and designating relatively cool and hot regions as a gradient of blue to red, respectively. We selected 13 red maples within the hottest regions and 13 within the coolest regions of the thermal map, each separated by more than 400 m (Figure 1a). Due to the mechanisms that cause urban sites to experience warmer and cooler temperatures, there are inherent differences in study site vegetation and impervious surface cover [23].

### Objective 1: Determine the effect of temperature on *M. tenebricosa* abundance

Thermal mapping was used for initial tree selection and gave relative site surface temperatures when the image was taken on 18 August 2007. To determine actual tree canopy temperatures, we placed iButton thermochron DS1921G (Dallas Semiconductor of Dallas, TX) remote temperature loggers within 59 ml portion containers (Dart Container Corporation Mason, MI) and fastened them to the underside of a branch within the canopy of each study tree. This method protected iButtons from direct sunlight and precipitation. iButtons recorded temperature every hour from April 2012 to April 2013. Due to loss of iButtons, complete 12-month temperature readings were only available for 22 of the 26 sites. Therefore, we used 7 months for which all sites had complete temperature data. These months in chronological order from 2012 to 2013 were: June, July, August, September, February, March, and April.

To measure *M. tenebricosa* abundance, we pruned one haphazardly selected 0.15 m terminal twig from four cardinal directions on each of our 26 study trees as described in [23]. To avoid biased selection, twigs were chosen based on length, presence of live foliage, and at a height of at least 3 meters at which point *M. tenebricosa* are not visually discernable. We collected twigs on four dates in 2012: 5 and 20 April, and 3 and 18 May. Twigs were examined under a dissecting microscope to record scale insect abundance per 0.6 m. In a previous study [23], we determined the importance of temperature in predicting scale insect abundance as it was influenced by several other exogenous variables in a path model. Here we were interested in the direct effect of temperature on scale insect abundance so we could then understand the effects of scale insect abundance and temperature on trees in Objectives 2 and 3. We tested the effect of mean 7-month tree canopy temperature on *M. tenebricosa* abundance using simple linear regression (JMP, Version 10. SAS Institute Inc., Cary, NC). Residuals of the simple regression of mean 7-month temperature predicting mean *M. tenebricosa* abundance followed a non-normal distribution (Shapiro-Wilk W = 0.902, P = 0.017). As such, scale insect abundance data were log_10_ (x+1) transformed to improve normality of the residuals. The resulting model followed the form:
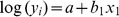
(1)where log(y_i_) is the observed response in log_10_ transformed *M. tenebricosa* abundance for the i^th^ tree; a is the y-intercept parameter to be estimated; b_1_ is the slope parameter to be estimated; and x_1_ is mean 7-month tree canopy temperature.

### Objective 2: Determine how temperature and *M. tenebricosa* abundance affect tree water potential and growth

Plant water potential is among the most accurate measures of plant moisture stress [29], [30]. It quantifies the amount of transpiration-driven negative pressure required to pull water up from the roots to the leaves [31]. Water-stressed plants require greater force to move water throughout their vascular system [31] and have a more negative water potential. Midday water potential is a measure of the peak stress level that a plant experiences during the day [29]. We measured midday water potential from noon to 3∶00 pm on 5, 7, 8, and 9 August 2013 with a pressure chamber (PMS Instrument Company, Albany, OR). Site sampling order was randomized. We collected one 25 cm twig with 10 to 30 fully expanded leaves from sun-exposed locations approximately 5.5 m above ground from the north and south side of each tree canopy. Weather conditions were similar for each day with a mean midday temperature of approximately 31°C, relative humidity of approximately 70% [32] and mostly sunny skies. We recorded xylem water potential within 5 minutes of twig abscission. We used multiple linear regression to determine how mean 7-month tree canopy temperature, *M. tenebricosa* abundance, and their interaction affect water potential (Eq. 2) in JMP 10.0. We also used multiple linear regression to test this hypothesis using two other temperature metrics in place of 7-month temperature to determine the temporal scale at which the urban environment affects tree stress: mean August temperature (Eq. 3) and midday temperature (Eq. 4) during the time of sampling. The resulting models were:
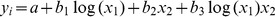
(2)

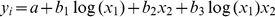
(3)

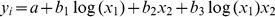
(4)where y_i_ is the observed response in mean water potential for the i^th^ tree; a is the y-intercept parameter to be estimated; b_i_ is the slope parameter to be estimated; log(x_1_) is log_10_ transformed mean *M. tenebricosa* abundance; x_2_ is mean 7-month tree canopy temperature (Eq. 2), mean August tree canopy temperature (Eq. 3), or mean midday tree canopy temperature (Eq. 4); and log(x_1_)x_2_ is the interaction term between temperature and *M. tenebricosa* abundance.

As a second evaluation of tree stress, we determined annual growth by measuring change in diameter at breast height (DBH). DBH measurements were conducted in accordance with National Forest Service guidelines [33] and taken at exactly 1.4 m above the highest ground on each study tree in June 2012 and July 2013. We calculated change in DBH as a measure of annual tree growth, which is reflective of tree vigor and carbon sequestration. Change in DBH was log_10_ (x+1) transformed to meet assumptions of linear regression analysis. In addition, change in DBH was dependent upon initial DBH. Therefore, we conducted a partial correlation by including the residuals of the correlation between initial DBH and change in DBH as a predictor variable. We used multiple linear regression to determine how mean seven-month temperature, *M. tenebricosa* abundance, and their interaction affect change in DBH over one year in JMP 10.0. The resulting model was:
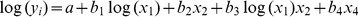
(5)where log(y_i_) is the observed response in log_10_ transformed change in tree DBH for the i^th^ tree; a is the y-intercept parameter to be estimated; b_i_ is the slope parameter to be estimated; log(x_1_) is log_10_ transformed mean *M. tenebricosa* abundance; x_2_ is mean 7-month tree canopy temperature; log(x_1_)x_2_ is the interaction term between temperature and *M. tenebricosa* abundance; and x_4_ is the residuals of the correlation between initial DBH and change in DBH.

Since change in DBH is often a slow progression of growth over a single year, we measured annual stem elongation as a historical assessment of resource allocation to above ground growth [34]. We randomly selected and pruned one twig from 8 sides of the canopy from each study tree. In late fall of 2013 we located and measured the distance from the apical tip to the most recent bud scar (2013 growth) and the distance from bud scar to previous bud scar, accounting for 2012 and 2011 growth. We then calculated mean annual growth over the past three years for each tree. We used multiple linear regression to determine how mean seven-month temperature, *M. tenebricosa* abundance, and their interaction affect mean annual stem elongation in JMP 10.0. The resulting model was:
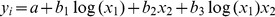
(6)where y_i_ is the observed response in 3-year mean annual stem elongation for the i^th^ tree; a is the y-intercept parameter to be estimated; b_i_ is the slope parameter to be estimated; log(x_1_) is log_10_ transformed mean *M. tenebricosa* abundance; x_2_ is mean 7-month tree canopy temperature; and log(x_1_)x_2_ is the interaction term between temperature and *M. tenebricosa* abundance.

### Objective 3: Determine how temperature and *M. tenebricosa* abundance affect tree condition

To qualitatively rate overall tree condition, each study tree was rated following the protocol used by the Raleigh Parks, Recreation, and Human Resources department, similar to that used by Berrang et al. [35]. These condition ratings are: Excellent, good, fair, poor, and dead. “Dead” trees were those with brittle branches and no live foliage but were not included in our study. “Poor” trees had many dead branches, broken branch tips, a broken central leader, exposed roots, and/or crispy leaves. “Fair” trees had some dead branches, wilted leaves, and less severe symptoms than poor trees. “Good” trees had no significant dead branches, no injuries, and may have sparse or coarse foliage. “Excellent” trees were defined as those with healthy foliage and full canopies. We recruited two volunteers in addition to a primary investigator to conduct tree condition evaluations on each of our study trees. For each tree, condition was recorded as the most common rating (shared by at least 2 of 3 observers). Using this method, we used multiple logistic regression to determine how seven-month temperature, scale insect abundance, and their interaction affect tree condition in JMP 10.0. The multiple logistic regression model was:
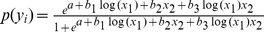
(7)Where *p(y_i_)* is the observed probability of a change in tree condition for the i^th^ tree as temperature and scale insect abundance change; a is the y-intercept parameter to be estimated; b_i_ is the slope parameter to be estimated; log(x_1_) is log_10_ transformed mean *M. tenebricosa* abundance; x_2_ is mean 7-month tree canopy temperature; and log(x_1_)x_2_ is the interaction term between temperature and *M. tenebricosa* abundance.

To determine if temperature affects street tree condition across the entire city, we analyzed tree condition ratings for 2780 city-owned red maple trees in Raleigh, NC. Starting with a tree inventory of over 8000 city-owned red maple trees created by the Raleigh Parks, Recreation, and Human Resources department (Figure 1b), we compared tree condition ratings to projected site surface temperatures. The thermal map projected surface temperature values representative of daily maximum August temperature at a 30×30 m resolution. In multiple instances, a 30×30 m thermal polygon contained more than one red maple tree. To prevent pseudo replication by selecting trees within the same polygon, we used ArcMap GIS software to randomly select one red maple tree within each discriminant polygon of the thermal map. We used Hawth’s tools for spatial ecological analysis to randomly select one tree within each temperature polygon containing one or more red maple trees. Interpolated site temperature values were then extracted at each selected point and compiled in an attribute table with corresponding tree condition ratings and DBH measurements. We selected trees to fall within the DBH range of our study trees (15.2 to 50.8 cm) to control for the effect of age on condition rating and to homogenize the inventory with our sample trees.

Due to a lack of fit in logistic regression and since Landsat surface temperature projections are discrete values, we classified temperature values into categorical variables for comparison with tree condition rating. Temperature values for the remaining 2780 trees were divided at the median (29.26°C) into “warm” and “cool” temperature classifications. The citywide data separated at the median represent a more conservative analysis of temperature effects on tree condition since they mostly include medium temperature trees. Because of this, we also classified the upper and lower quartile of the citywide distribution as “hot” and “cold”, respectively. This gives a more similar representation of our subsample and shows temperature extremes that will become more common as urban warming couples with global climate change. “Excellent” and “poor” ratings were compared between temperature classifications because they were the most confidently rated tree conditions. We conducted contingency analyses and Pearson’s chi square tests to test the hypothesis that tree condition is dependent on site temperature using JMP 10.0.

## Results

### Objective 1: Determine how temperature affects *M. tenebricosa* abundance

**Figure 2:**
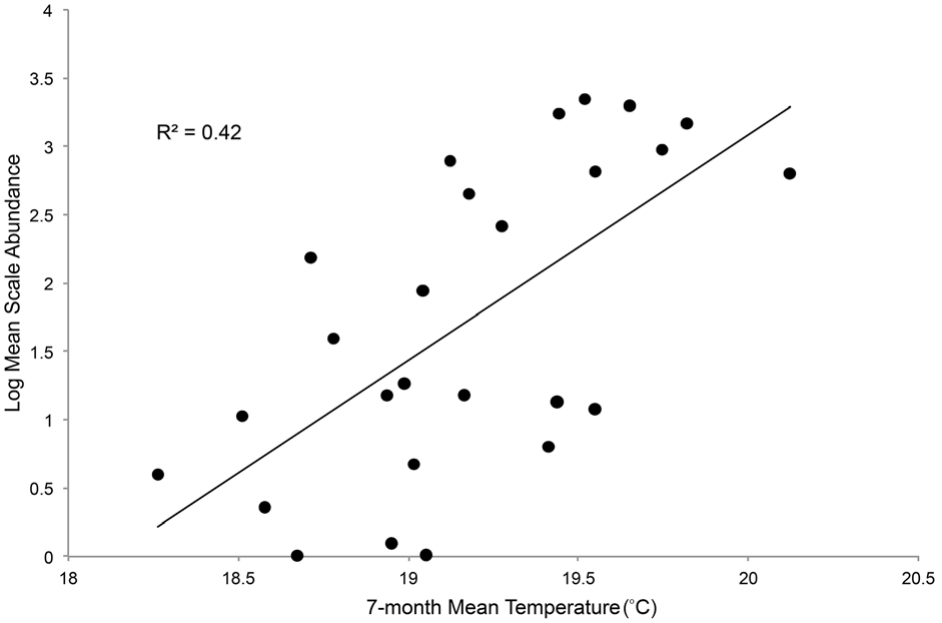
Linear regression of seven-month mean temperature and log mean scale abundance per 0.6 m of maple twig (log(y) = −29.95+1.65x). Regression model is statistically significant (P<0.05).

Mean *M. tenebricosa* abundance included all life stages and ranged from 0 to 2241 live individuals per 0.6 m of twig with a mean (±SD) of 444 (±683.66). Seven-month mean tree canopy temperature ranged from 18.3 to 20.1°C with a mean of 19.2°C (±0.44). Mean log *M. tenebricosa* abundance per 0.6 m of twig was significantly (alpha = 0.05), positively correlated with seven-month mean tree canopy temperature (Figure 2, Table 1).

**Table 1:** Parameter estimates table of multiple regression analyses.

		R^2^	F	df	Estimate	SE	t	P	VIF
Scale abund.*		0.42	17.12	24				**0.0004**	
	Intercept			24	−29.95	7.66	−3.91	**0.0007**	
	7-month temp.			24	1.65	0.40	4.14	**0.0004**	
Midday water potential		0.19	1.68	22				0.199	
	Intercept			22	11.31	17.75	0.64	0.531	
	Scale abund.*			22	0.58	1.04	0.56	0.583	2.52
	Midday temp.			22	0.34	0.61	0.55	0.588	2.62
	Interaction			22	−0.51	0.40	−1.28	0.214	1.09
Midday water potential		0.49	7.10	22				**0.0016**	
	Intercept			22	−66.08	25.02	−2.64	**0.015**	
	Scale abund.*			22	−0.42	0.71	−0.58	0.565	1.84
	August temp.			22	3.55	1.02	3.47	**0.002**	1.90
	Interaction			22	−1.49	0.76	−1.97	*0.062*	1.04
Midday water potential		0.36	4.09	22				**0.0189**	
	Intercept			22	−61.2	36.47	−1.68	0.107	
	Scale abund.*			22	0.09	0.76	0.12	0.906	1.71
	7−month temp.			22	4.38	1.94	2.25	**0.035**	1.73
	Interaction			22	−3.27	1.62	−2.01	*0.056*	1.02
Change in DBH		0.92	62.76	21				**<0.0001**	
	Intercept			21	−0.94	0.38	−2.46	**0.023**	
	Scale abund.*			21	0.01	0.008	1.41	0.174	1.81
	7-month temp.			21	0.06	0.02	2.80	**0.011**	1.74
	Interaction			21	−0.03	0.02	−1.70	0.105	1.04
	Residuals Initial by change in dbh			21	0.97	0.07	14.08	**<0.0001**	1.06
3-yr annual stem elongation		0.09	0.69	22				0.567	
	Intercept			22	17.72	48.02	0.37	0.716	
	Scale abund.*			22	1.10	1.01	1.09	0.287	1.71
	7-month temp.			22	−0.49	2.56	−0.19	0.850	1.73
	Interaction			22	1.02	2.14	0.48	0.637	1.02

*Scale abund. refers to log_10_ transformed *M. tenebricosa* abundance; significant results are bolded.

R^2^ = coefficient of determination; F = F-statistic; df = degrees of freedom; Estimate = parameter estimate; SE = standard error; t = t-statistic; P = p-value; VIF = Variance inflation factor.

### Objective 2: Determine how temperature and *M. tenebricosa* abundance affect tree water potential and growth

**Figure 3:**
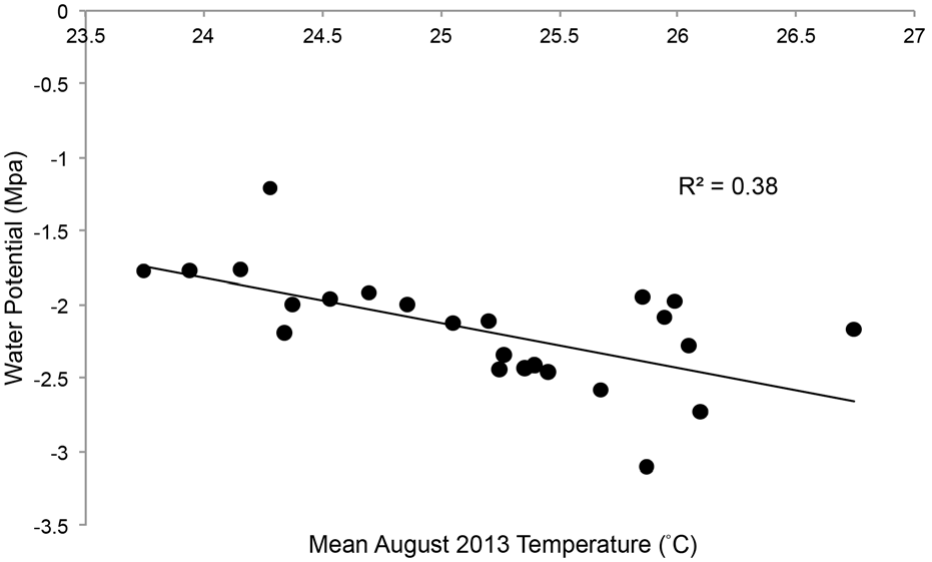
Linear regression of mean August 2013 tree canopy temperature and mean stem water potential (MPa) (y = 5.53–0.31x). Regression model is statistically significant (P<0.05).

Mean midday stem water potential ranged from −1.21 to −3.10 MPa with a mean (±SD) of −2.19 MPa (±0.39). Multiple linear regression revealed no association between midday tree canopy temperature and mean tree water potential, mean *M. tenebricosa* abundance, or their interaction (Table 1). Based on multiple linear regression, mean August temperature was significantly associated with mean water potential but *M. tenebricosa* abundance was not (Figure 3, Table 1). There was a nearly significant interaction between mean *M. tenebricosa* abundance and mean August temperature in predicting mean water potential (Table 1), suggesting that the effect of temperature on water potential may not be independent of scale insect abundance. Multiple linear regression using mean 7-month temperature revealed a significant association with mean water potential, while mean *M. tenebricosa* abundance was not (Table 1). As temperature increased, mean water potential significantly decreased. There was also a nearly significant interaction between 7-month temperature and *M. tenebricosa* abundance in predicting water potential (Table 1). Variance inflation factors (VIF) for each tested model did not suggest an effect of collinearity on the output (VIF<3).

Change in DBH ranged from 0 to 4.3 cm with a mean (±SD) of 1.3 cm (±1.08). The full model predicting change in DBH was significant and explained approximately 91% of the variation in the dataset (Table 1). Change in DBH was positively associated with mean 7-month tree canopy temperature, but we detected no effect of *M. tenebricosa* abundance, or its interaction with temperature (Table 1). Although collinearity is a potential influence, VIF values for the full model did not suggest an effect of collinearity on the results (VIF<2). Three-year mean annual stem elongation ranged from 0.08 to 0.49 m with a mean of 0.27 m (±0.11) and was not associated with mean 7-month tree canopy temperature, mean *M. tenebricosa* abundance, or their interaction (Table 1).

### Objective 3: Determine how temperature and *M. tenebricosa* abundance affect tree condition

**Figure 4:**
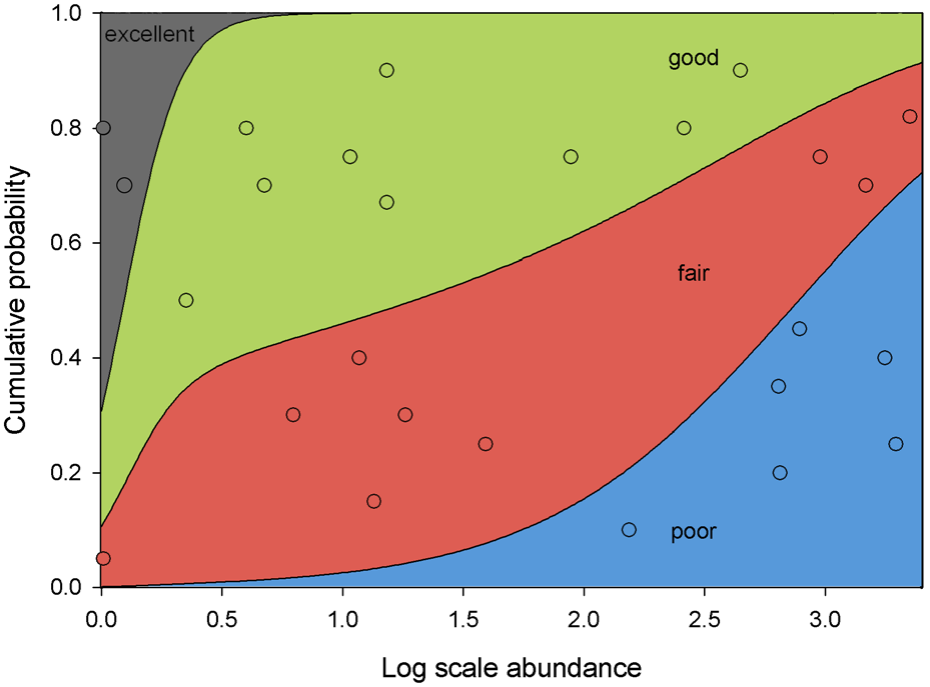
Since scale insect abundance was the most important predictor in our multiple logistic regression model, we use simple logistic regression to illustrate the relationship between scale insect abundance and tree condition. Points represent recorded measures of *M. tenebricosa* abundance on the x-axis but are not associated with y-axis cumulative probabilities. Colored sections are associated with the cumulative probabilities on the y-axis as they change across measured *M. tenebricosa* abundance on the x-axis.

Landsat surface temperatures correlated well with 7-month mean iButton tree canopy temperatures (r = 0.76), supporting our use of Landsat surface temperature as a proxy for tree canopy temperature. The full logistic regression model predicting tree condition was significant and explained 44% of the variation in the model (Table 2). *Melanaspis tenebricosa* abundance was significantly associated with tree condition, while seven-month temperature and the interaction between the two were not (Table 2). As *M. tenebricosa* abundance increased, the probability of finding a tree in poor condition significantly increased while the probability of finding a tree in excellent condition significantly decreased (Figure 4). These results suggest that temperature does not contribute any additional value to predicting the variation in tree condition that *M. tenebricosa* is able to predict on its own.

**Table 2:** Parameter estimate table of multiple logistic regression predicting tree condition.

		R^2^	df	Estimate	SE	*χ* ^2^	P
Tree Condition		0.44	9			29.13	**0.0006**
	Intercept		9	−21.76	71.56	0.09	0.761
	*M. tenebricosa* abund.		9	−2.54	1.29	3.90	**0.048**
	7-month temp.		9	1.43	3.75	0.15	0.702
	*M. tenebricosa* abund.×7-month temp.		9	1.46	3.47	0.18	0.674

*Significant results are bolded.

**Figure 5:**
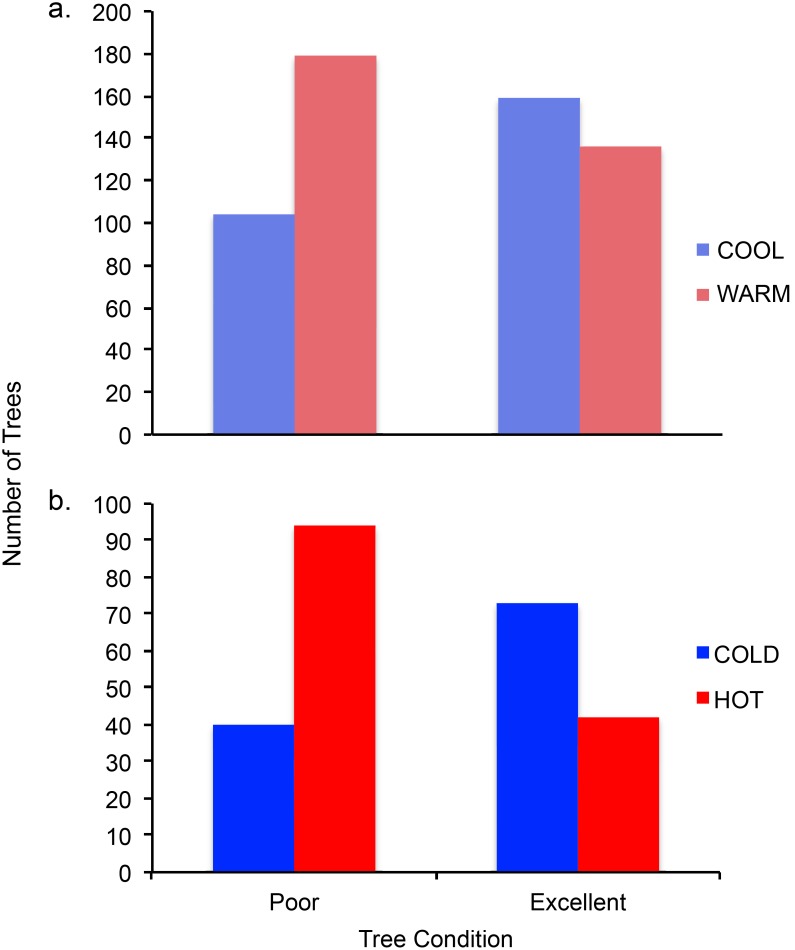
Pairwise comparison (a) of the number of trees with “Poor” and “Excellent” condition ratings between “Warm” and “Cool” median temperature classification (P<0.0001). Pairwise comparison (b) of the number of trees with “Poor” and “Excellent” condition ratings between “Hot” and “Cold” quartile temperature classification (P<0.0001).

Global contingency analysis of tree condition and site temperature divided at the median showed that there was a significant difference in the likelihood of tree condition rating between warm and cool sites (Table 3). Pairwise comparison of excellent and poor condition trees revealed that trees in warm sites are significantly more likely to be in poor condition than those in cool sites (Figure 5a, Table 3). In addition, over 63% of trees in poor condition were located in warm sites (Table S1).

**Table 3:** Pearson Chi-squared test.

Tree condition division		N	df	Pearson χ^2^	P
Median					
	Global	2780	4	24.09	**<0.0001**
	Pairwise	578	1	17.13	**<0.0001**
Upper & lower quartiles					
	Global	1397	4	32.39	**<0.0001**
	Pairwise	249	1	28.23	**<0.0001**

*Significant results are bolded.

The upper quartile included sites greater than or equal to 30.68°C and lower quartile sites were less than or equal to 27.74°C. Global contingency analysis of these data also showed a significant difference in the likelihood of tree condition ratings between hot and cold sites (Table 3). Pairwise comparison of excellent and poor condition trees revealed that trees in hot sites were significantly more likely to be in poor condition when compared to those in cold sites (Figure 5b, Table 3). Hot sites had over twice as many trees in poor condition than cold sites (Table S1). Detecting such a difference in both median and quartile analyses further supports the suggestion that temperature is associated with tree condition.

## Discussion

Many herbivorous pests including lacebugs [36], mites [37], caterpillars [10], soft scales [22], and other armored scales [38], [39] are more abundant and damaging in urban than rural habitats [5], [7], [8], [40]. Although the mechanisms underlying this phenomenon are not well understood [8], mounting evidence points to high urban temperatures as an important factor [22], [23]. We acknowledge that our non-probability sampling method limits the scope of inference from our sample to the total street tree population. Despite this limitation, our results suggest that urban warming increases scale insect abundance on street trees (Figure 2). Moreover, our results suggest that warming and scale insect abundance each contribute to reducing red maple street tree condition. Meineke et al. [22] found that a soft scale insect was more abundant on warmer than cooler street trees due to an increase in scale insect survival. In a previous study, we found that *M. tenebricosa* in warmer urban habitats are more fecund than those in cooler urban habitats, leading to greater population growth rates [23]. Greater survival, fecundity, and potentially other direct effects of temperature contribute to greater herbivore abundance on warmer street trees.

Heat can induce drought stress in trees by reducing soil moisture content and increasing vapor pressure deficits [17], [41], which leads to a greater demand for transpiration [18], [19] but also stomata closure and lower photosynthesis rates [21]. In addition, impervious surfaces replace soil volume that tree roots would otherwise exploit for water and nutrient uptake. As predicted, we found that trees in warmer urban habitats were significantly more water stressed than those in cooler urban habitats (Figure 3). Although not addressed in this paper, impervious surfaces and urban habitats also create conditions such as soil compaction that can increase water stress [42]. Such conditions are likely also worsened by warmer temperatures. Water stress can have negative effects on tree health by causing xylem embolism and subsequent cavitation within vascular tissue, which can reduce tree condition [43]. We have determined that scale insect abundance is positively associated with temperature but our results may suggest that it is also influenced by water potential (Table 1). Such a relationship is supported by Cockfield and Potter [24] who found a strong interaction between plant water stress and scale insect abundance on euonymus plants, as well as Hanks and Denno [38] who found that street tree water stress negatively affected the fitness of an armored scale insect.

In some cases plant stress can lead to greater herbivore abundance [21], [44]–[46]. For example, White [45] demonstrated that stressed plants circulate greater free amino acid content throughout their vascular system, which herbivores are able to exploit during feeding. However, this primarily applies to foliar-feeding insects [45]. Plant water stress most often reduces the fitness of sap feeding insects [24], [47], [48]. Hanks and Denno [38] found that armored scale insect survival and abundance declined with increasing water stress on street trees. *Melanaspis tenebricosa*, in addition to other armored scale insects, feed on parenchyma cells within the cambium of trees, which does not provide direct access to vascular circulation as in other sap-feeding insects [28], [49]. The mechanism behind the plant stress – herbivore relationship in our system remains unknown but is an important area for future research and may explain some of the remaining variation in *M. tenebricosa* abundance.

Warmer urban temperatures are often associated with less vegetation cover and greater sun exposure [23], [36]. These conditions can increase tree growth rates by increasing photosynthesis and carbon sequestration, especially when ample water and nutrients are available [50]. Despite greater water stress at warmer temperatures, we found a significant increase in DBH growth rate as tree canopy temperature increased (Table 1), suggesting that conditions were below that which negatively affects growth. Spring and summer 2013 were also abnormally wet, with rainfall approximately 34% above normal [32]. We suspect that this drastic increase in water availability may have reduced water stress and temperature compared to other years, allowing growth to continue where conditions would otherwise be limiting. In addition, DBH is only one component of growth and may not be representative of overall tree condition, especially at a scale as small as we found, and among trees of different ages. Stem elongation is extremely variable and dependent on sunlight exposure, competition for dominance among stems, and position within the canopy [51]. In addition, plants in poor health or that have lost tissue to herbivores sometimes compensate with greater growth on the remaining tissue [52]. Since *M. tenebricosa* causes branch dieback, trees with more scales may have fewer branches than cool trees but equal growth in the branches they do have.

We found that scale insect abundance and significant reductions in tree condition coincide with a less than 2°C increase in temperature (Figure 2, Figure 4), which global warming is predicted to exceed within this century [53]–[55]. Heat and scale insect abundance each contributed to reduce the condition of red maples. Although trees in poor condition comprised only 10% of red maple street trees, they were significantly more likely to be in warmer urban sites, which based on our results, may have had more *M. tenebricosa*. This difference was even significant when temperatures were divided at the median, where half of the trees were at medium temperatures. Our quartile analysis shows that the upper temperature extremes have over twice as many trees in poor condition than cooler sites, which represent conditions that may become more common as urban and global warming progress. Moreover, nearly 90% of red maple street trees are in less than excellent condition, which may reflect the unsuitability of urban habitats for red maple trees.

Management of urban forests is important but difficult due to the diversity of plants, organisms that attack them, and the conditions in which trees are planted. Different tree species have different habitat requirements based on provenance and evolutionary histories. Therefore, some trees are more suitable for street tree plantings than others. In addition, our results and others [22], [23], [36], [39] illustrate that certain areas within cities are more prone to greater herbivore abundance and damage. Although our study is limited to one tree species, our results suggest that satellite images, tree inventories, and GIS methods similar to ours can be useful for selecting tree species and suitable planting sites. As urbanization and climate change progress, such techniques may be critical to sustaining and managing productive urban forests.

## Supporting Information

Figure S1 (Left) Red maple heavily infested with *M. tenebricosa* exhibiting branch dieback and darkened bark. (Right) Red maple branch moderately infested with *M. tenebricosa* (bumps on bark).
(JPG)

Table S1 **Contingency table comparing tree condition to surface temperature division.**
(DOCX)

## References

[1] Oke TR, Crowther JM, McNaughton KG, Monteith JL, Gardiner B (1989) The Micrometeorology of the Urban Forest [and Discussion]. Philosophical Transactions of the Royal Society B: Biological Sciences 324: 335–349.

[2] Pataki DE, Alig RJ, Fung AS, Golubiewski NE, Kennedy CA, et al (2006) Urban ecosystems and the North American carbon cycle. Global Change Biology 12: 2092–2102.

[3] Donovan GH, Butry DT, Michael YL, Prestemon JP, Liebhold AM, et al (2013) The relationship between trees and human health: evidence from the spread of the emerald ash borer. Am J Prev Med 44: 139–145. 23332329 10.1016/j.amepre.2012.09.066

[4] Boyd IL, Freer-Smith PH, Gilligan CA, Godfray HC (2013) The consequence of tree pests and diseases for ecosystem services. Science 342: 1235773. 24233727 10.1126/science.1235773

[5] Putnam JD (1880) Biological and other notes on Coccidae. Proceedings of the Davenport Academy of Sciences 2: 293.

[6] Metcalf ZP (1912) The gloomy scale, an important enemy of shade trees in North Carolina. Journal of the Elisha Mitchell Scientific Society 28: 88–91.

[7] Metcalf ZP (1922) The gloomy scale. NC Agriculture Experiment Station Technical Bulletin 21: 1–23.

[8] Raupp MJ, Shrewsbury PM, Herms DA (2010) Ecology of herbivorous arthropods in urban landscapes. Annu Rev Entomol 55: 19–38. 19961321 10.1146/annurev-ento-112408-085351

[9] Kim HH (1992) Urban heat island. International Journal of Remote Sensing 13: 2319–2336.

[10] Coffelt MA, Schultz PB (1993) Larval parasitism of orangestriped oakworm (Lepidoptera: Saturniidae) in the urban shade tree environment. Biological Control 3: 127–134.

[11] Cockfield SD, Potter DA, Houtz RL (1987) Chlorosis and reduced photosynthetic CO2 assimilation of Euonymus fortunei infested with euonymus scale (Homoptera: Diaspididae). Environ Entomol 16: 1314–1318.

[12] Schaffer B, Mason LJ (1990) Effects of scale insect herbivory and shading on net gas exchange and growth of a subtropical tree species (Gualacum sanctum L.). Oecologia 84: 468–473. 28312962 10.1007/BF00328162

[13] Vranjic JA, Ash JE (1997) Scale insects consistently affect roots more than shoots: The impact of infestation size on growth of Eucalypt seedlings. Journal of Ecology 85: 143–149.

[14] Brightwell RJ, Silverman J (2009) Invasive Argentine ants reduce fitness of red maple via a mutualism with an endemic coccid. Biological Invasions 12: 2051–2057.

[15] Zvereva EL, Lanta V, Kozlov MV (2010) Effects of sap-feeding insect herbivores on growth and reproduction of woody plants: a meta-analysis of experimental studies. Oecologia 163: 949–960. 20397030 10.1007/s00442-010-1633-1

[16] Oke TR (1973) City size and the urban heat island. Atmospheric Environment 7: 769–779.

[17] Jenerette GD, Scott RL, Barron-Gafford GA, Huxman TE (2009) Gross primary production variability associated with meteorology, physiology, leaf area, and water supply in contrasting woodland and grassland semiarid riparian ecosystems. Journal of Geophysical Research 114.

[18] Whitlow TH, Bassuk NL (1988) Ecophysiology of urban trees and their management - The North American experience. Hortscience 23: 542–546.

[19] Álvarez S, Navarro A, Nicolás E, Sánchez-Blanco MJ (2011) Transpiration, photosynthetic responses, tissue water relations and dry mass partitioning in Callistemon plants during drought conditions. Scientia Horticulturae 129: 306–312.

[20] Katul G, Leuning R, Oren R (2003) Relationship between plant hydraulic and biochemical properties derived from a steady-state coupled water and carbon transport model. Plant, Cell, and Environment 26: 339–350.

[21] McDowell N, Pockman WT, Allen CD, Breshears DD, Cobb N, et al (2008) Mechanisms of plant survival and mortality during drought: why do some plants survive while others succumb to drought? New Phytol 178: 719–739. 18422905 10.1111/j.1469-8137.2008.02436.x

[22] Meineke EK, Dunn RR, Sexton JO, Frank SD (2013) Urban warming drives insect pest abundance on stree trees. PlosOne 8. 10.1371/journal.pone.0059687 PMC3609800 23544087

[23] Dale AG, Frank SD (In press) Urban warming trumps natural enemy regulation of herbivorous pests. Ecological Applications. 10.1890/13-1961.1 29210225

[24] Cockfield SD, Potter DA (1986) Interaction of Euonymus scale (Homoptera: Diaspididae) damage and severe water stress on leaf abscission and growth of Euonymus fortunei. Oecologia 71: 41–46. 28312081 10.1007/BF00377318

[25] Frank SD, Klingeman WE, White SA, Fulcher A (2013) Biology, Injury, and Management of Maple Tree Pests in Nurseries and Urban Landscapes. Journal of Integrated Pest Management 4: 1–14.

[26] Raupp MJ, Cumming AB, Raupp EC (2006) Street tree diversity in eastern North America and its potential for tree loss to exotic borers. Arboriculture & Urban Forestry 32: 297–304.

[27] Nesom G (2006) Plant guide: Red maple. In: USDA, editor. Natural resources conservation service national plant data center and the biota of North America program.

[28] Beardsley JW, Gonzalez RH (1975) The biology and ecology of armored scales. Annual Review of Entomology 20: 47–73. 10.1146/annurev.en.20.010175.000403 1090245

[29] McCutchan H, Shackel KA (1992) Stem-water potential as a sensitive indicator of water stress in prune trees (Prunus domestica L. cv. French). Journal of the American Society of Horticultural Science 117: 607–611.

[30] De Swaef T, Steppe K, Lemeur R (2009) Determining reference values for stem water potential and maximum daily trunk shrinkage in young apple trees based on plant responses to water deficit. Agricultural Water Management 96: 541–550.

[31] Scholander PF, Hammel HT, Bradstreet ED, Hemmingsen EA (1965) Sap pressure in vascular plants. Science 148. 10.1126/science.148.3668.339 17832103

[32] State Climate Office of North Carolina (2013) Daily and monthly relative humidity and precipitation data. In: Cronos database.

[33] USDA Forest Service (2007) Forest inventory and analysis national core field guide: Field data collection procedures for phase 2 plots. In: National core field guide. 69–78.

[34] Johns RC, Leggo JJ, MacLean DA, Quiring DT (2013) Relationships between Pikonema alaskensis larval density and shoot growth and production in young black spruce. Forest Ecology and Management 292: 130–138.

[35] Berrang P, Karnosky DF, Stanton J (1985) Environmental factors affecting tree health in New York City. Journal of Arboriculture 11: 185–189.

[36] Shrewsbury PM, Raupp MJ (2000) Evaluation of Components of Vegetational Texture for Predicting Azalea Lace Bug, *Stephanitis pyrioides* (Heteroptera: Tingidae), Abundance in Managed Landscapes. Environmental Entomology 29: 919–926.

[37] Kropczynska D, van de Vrie M, Tomczyk A (1986) Woody ornamentals. In: Helle W, Sabelis MW, editors. Spider mites, their biology, natural enemies and control: Amsterdam: Elsevier. 385–393.

[38] Hanks LM, Denno RF (1993) Natural enemies and plant water relations influence the distribution of an armored scale insect. Ecology 74: 1081–1091.

[39] Tooker JF, Hanks LM (2000) Influence of Plant Community Structure on Natural Enemies of Pine Needle Scale (Homoptera: Diaspididae) in Urban Landscapes. Environmental Entomology 29: 1305–1311.

[40] Cuevas-Reyes P, Gilberti L, González-Rodríguez A, Fernandes GW (2013) Patterns of herbivory and fluctuating asymmetry in Solanum lycocarpum St. Hill (Solanaceae) along an urban gradient in Brazil. Ecological Indicators 24: 557–561.

[41] Cregg BM, Dix ME (2001) Tree moisture stress and insect damage in urban areas in relation to heat island effects. Journal of Arboriculture 27: 8–17.

[42] Smiley ET, Calfee L, Fraedrich BR, Smiley EJ (2006) Comparison of structural and noncompacted soils for trees surrounded by pavement. Arboriculture & Urban Forestry 32: 164–169.

[43] Sperry J, Tyree MT (1988) Mechanism of water stress-induced xylem embolism. Plant Physiology 88: 581–587. 16666352 10.1104/pp.88.3.581 PMC1055628

[44] Mattson WJ (1980) Herbivory in relation to plant nitrogen content. Annual Review of Ecology and Systematics 11: 119–161.

[45] White TCR (1984) The abundance of invertebrate herbivores in relation to the availability of nitrogen in stressed food plants. Oecologia 63: 90–105. 28311171 10.1007/BF00379790

[46] Miller TEX, Tyre AJ, Louda SM (2006) Plant reproductive allocation predicts herbivore dynamics across spatial and temporal scales. The American Naturalist 168: 608–616. 10.1086/509610 17080360

[47] Koricheva J, Larsson S (1998) Insect performance on experimentally stressed woody plants: A meta-analysis. Annual Review of Entomology 1998. 10.1146/annurev.ento.43.1.195 15012389

[48] Huberty AF, Denno RF (2004) Plant water stress and its consequences for herbivorous insects: A new synthesis. Ecology 85: 1383–1398.

[49] Miller DR, Davidson JA (2005) Armored scale insect pests of trees and shrubs (Hemiptera: Diaspdidae). Comstock publishing associates: Cornell University Press, Ithaca and London.

[50] Way DA, Oren R (2010) Differential responses to changes in growth temperature between trees from different functional groups and biomes: a review and synthesis of data. Tree Physiol 30: 669–688. 20368338 10.1093/treephys/tpq015

[51] Lambers H, Chapin III FS, Pons TL (1998) Growth and allocation. Plant physiological ecology. Berlin: Springer-Verlag. 299–351.

[52] McNaughton SJ (1983) Compensatory plant growth as a response to herbivory. Oikos 40: 329–336.

[53] Christensen JH, Hewitson B, Busuioc A, Chen A, Gao X, et al.. (2007) Regional Climate Projections. In: Climate Change 2007: The Physical Science Basis. Contribution of Working Group I to the Fourth Assessment Report of the Intergovernmental Panel on Climate Change. Cambridge University Press, Cambridge, United Kingdom and New York, NY, USA.

[54] Meehl GA, Stocker TF, Collins WD, Friedlingstein P, Gaye AT, et al. (2007) Global Climate Projections. In: Solomon S, Qin D, Manning M, Chen Z, Marquis M, et al.., editors. Climate Change 2007: The physical science basis Contribution of working group I to the fourth assessment report of the intergovernmental panel on climate change. Cambridge, United Kingdom and New York, NY, USA: Cambridge University Press.

[55] Hansen J, Ruedy R, Sato M (2010) Global surface temperature change. Review of Geophysics 48: 1–29.

