# Supplementary figures and images for: The Effects of Urban Warming on Herbivore Abundance and Street Tree Condition
Source: PLoS One. 2014 Jul 23;9(7):e102996. doi: 10.1371/journal.pone.0102996 (PMC4108386; doi:10.1371/journal.pone.0102996)

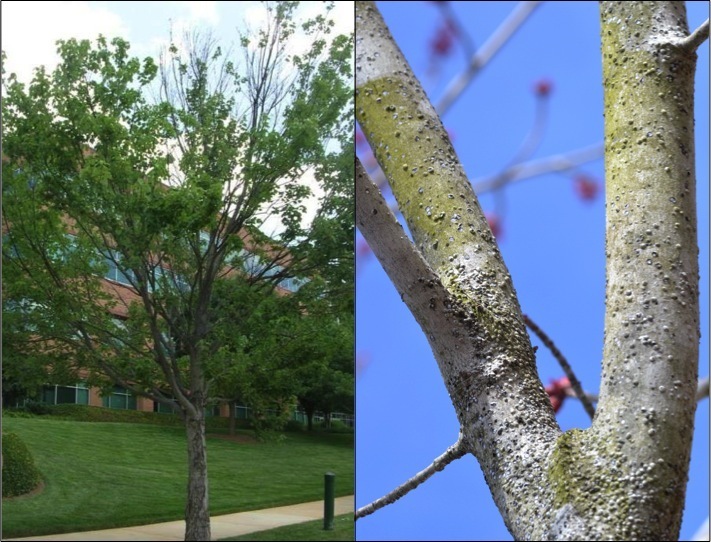

Supplement: Figure S1 — (Left) Red maple heavily infested with M. tenebricosa exhibiting branch dieback and darkened bark. (Right) Red maple branch moderately infested with M. tenebricosa (bumps on bark). (JPG) [file pone.0102996.s001.jpg]
