# Supplementary material for: The Effects of Urban Warming on Herbivore Abundance and Street Tree Condition
Source: PLoS One. 2014 Jul 23;9(7):e102996. doi: 10.1371/journal.pone.0102996 (PMC4108386; doi:10.1371/journal.pone.0102996)
Supplement: Table S1 — Contingency table comparing tree condition to surface temperature division. (DOCX) [file pone.0102996.s002.docx]

| **Table S1.** Contingency table of tree condition | | | |
| --- | --- | --- | --- |
| Count  Column %  Row % |  | Poor | Excellent |
| Median division |  |  |  |
|  | Warm | 179  63.25  56.83 | 136  46.10  43.17 |
|  | Cool | 104  36.75  39.54 | 159  53.90  60.46 |
| Upper & lower quartile division |  |  |  |
|  | Hot | 94  70.15  69.12 | 42  36.52  30.88 |
|  | Cold | 40  29.85  35.40 | 73  63.48  64.60 |
